# Genetic diversity of HPV35 in Chad and the Central African Republic, two landlocked countries of Central Africa: A cross-sectional study

**DOI:** 10.1371/journal.pone.0297054

**Published:** 2024-01-25

**Authors:** Ralph-Sydney Mboumba Bouassa, Juval Avala Ntsigouaye, Paola Candyse Lemba Tsimba, Zita Aleyo Nodjikouambaye, Damtheou Sadjoli, Marcel Mbeko Simaleko, Serge Police Camengo, Jean De Dieu Longo, Gérard Grésenguet, David Veyer, Hélène Péré, Christian Diamant Mossoro-Kpinde, Laurent Bélec

**Affiliations:** 1 Laboratoire de virologie, Hôpital Européen Georges Pompidou, Assistance Publique-Hôpitaux de Paris (AP-HP) and Université Paris Cité, Paris, France; 2 Ecole Doctorale Régionale (EDR) d’Afrique Centrale en Infectiologie Tropicale, Franceville, Gabon; 3 Faculté des Sciences de la Santé, Université Marien Ngouabi, Brazzaville, Republic of the Congo; 4 Service de Gynécologie-Obstétrique, Hôpital de la Mère et de l’Enfant, N’Djamena, Chad; 5 Cabinet Médical de Gynécologie Obstétrique "La Renaissance Plus," N’Djamena, Chad; 6 Centre National de Référence des Infections Sexuellement Transmissibles et de la Thérapie Antirétrovirale, Bangui, Central African Republic; 7 Service de Gastro-entérologie, Hôpital de l’Amitié, Bangui, Central African Republic; 8 Faculté des Sciences de la Santé, Université de Bangui, Bangui, Central African Republic; 9 Unité de Recherches et d’Intervention sur les Maladies Sexuellement Transmissibles et le SIDA, Département de Santé Publique, Bangui, Central African Republic; Cairo University Faculty of Veterinary Medicine, EGYPT

## Abstract

Human Papillomavirus (HPV)-35 accounts for up 10% of cervical cancers in Sub-Saharan Africa. We herein assessed the genetic diversity of HPV35 in HIV-negative women from Chad (identified as #CHAD) and HIV-infected men having sex with men (MSM) in the Central African Republic (CAR), identified as #CAR. Ten HPV35 DNA from self-collected genital secretions (n = 5) and anal margin samples (n = 5) obtained from women and MSM, respectively, were sequenced using the ABI PRISM^®^ BigDye Sequencing technology. All but one HPV35 strains belonged to the A2 sublineage, and only #CAR5 belonged to A1. HPV35 from #CAR had higher L1 variability compared to #CHAD (mean number of mutations: 16 *versus* 6). L1 of #CAR5 showed a significant variability (2.29%), suggesting a possible intra-type divergence from HPV35H. Three (BC, DE, and EF) out of the 5 capsid loops domains remained totally conserved, while FG- and HI- loops of #CAR exhibited amino acid variations. #CAR5 also showed the highest LCR variability with a 16bp insertion at binding sites of the YY1. HPV35 from #CHAD exhibited the highest variability in E2 gene (*P*<0.05). E6 and E7 oncoproteins remained well conserved. There is a relative maintenance of a well conserved HPV35 A2 sublineage within heterosexual women in Chad and MSM with HIV in the Central African Republic.

## Introduction

High risk-human papillomavirus (HR-HPV) are the main etiology for both cervical and anal cancers in women and men, with HPV16 and HPV18 causing more than 70% of cervical cancers and 90% of anal cancers worldwide [1–6]. With almost 117,000 news cases diagnosed and up to 77,000 deaths recorded in 2020, cervical cancer ranks the second most diagnosed and the leading cause of cancer death in women in sub-Saharan Africa (SSA) [7]. HPV-associated anal cancer in men in SSA remains poorly documented but is expected to be frequent, especially in men having sex with men (MSM), given the high prevalence in this population of both anal HR-HPV and HIV, the two main risk factors for anal cancer in MSM [8–12].

Although HPV16 is considered the most detected genotype in SSA [13, 14], there is a growing body of literature showing that HPV35 is also highly prevalent in SSA in both women [15–22] and MSM [11, 23], particularly in people living with HIV [10, 15, 16, 23–31]. For instance, we have previously shown that HPV35 was the most frequently detected genotype in MSM living in the Central African Republic (CAR) [10], and the second most detected genotype after HPV58, in adult women living in Chad [30]. This genotype, the phylogenetically closest to HPV16, accounts for only ~2% of the worldwide burden of cervical cancer [4, 32, 33], but this rate is increased by up to 5-fold in SSA [13, 19, 22, 33–36], making HPV35 one of the most important but neglected HR-HPV in SSA [13, 22]. Note that, unfortunately, the currently commercially available HPV vaccines are not targeting HPV35 [2, 37]. Like what is observed for HPV16 [38–41], genetic variations and host-specific adaptation would likely explain this unique distribution of HPV35 and its attributable cancer burden [33]. Indeed, recent studies conducted on women living in SSA or those with African ancestry uncovered an association between cervical cancer and the A2 sublineage of HPV35 [33, 42]. Another study on mixed ethnicity women showed that the A1 sublineage of HPV35 was significantly more associated with cervical cancer than the A2 [43]. On the other hand, no data exist regarding the impact of the genetic variability of HPV35 in anal cancer in African MSM. Given the critical role of HPV35 in the burden of cervical cancers in SSA and its high prevalence in both heterosexual women and MSM in this continent, it is therefore necessary to further study the genetic variability of HPV35 circulating in these populations. As stated above, HPV35 was the second most frequently detected genotype in women (10.3%; 6/58) living in Chad and the most detected in MSM (27.6%; 8/29) in CAR [10, 30]. Adding to the canon of global HPV sequence data is worthwhile, particularly from regions of the world where particular genotypes with underestimated oncogenic potential thus far may be predominant. Herein, we thus aimed to explore the genetic variability of clinical strains of HPV35 isolated from HIV-negative heterosexual adult women living in N’Djamena, in Chad [30] and HIV-infected MSM living in CAR [10], two African populations in whom HPV35 has been previously found to be highly prevalent.

## Material and methods

### Study design and population

We recently conducted descriptive cross-sectional surveys estimating the prevalence of cervical and anal HR-HPV infections and associated risk factors among adult heterosexual women attending the clinic for women’s sexual health “La Renaissance Plus” in N’Djamena, Chad, recruited in 2018 (so-called ***Gyn-Auto study***) [30] and MSM receiving care recruited during 2010 to 2018, at the Centre National de Référence des Infections Sexuellement Transmissibles et de la Thérapie Antirétrovirale (CNRIST/TAR) of Bangui, the capital city of the CAR (so-called ***HomoPap study***) [10], respectively.

For the present study, adult Chadian heterosexual women included in the ***Gyn-Auto study*** [30] and adult Central African MSM included in the ***HomoPap study*** [10], and having been diagnosed positive for HPV-35 DNA by Nucleic-Acid Amplification Test (NAAT) were included. Women underwent a cervical cytology via Papanicolaou-stained conventional smear, as recommended by the 2021-revised WHO guidelines [44], and interpreted by pathologists according to the 2001 Bethesda system [44]. Similarly, a physician performed a clinical exploration of the perianal area and of the anal orifice of MSM attending the CNRIST/TAR via a digital rectal examination or a high-resolution anuscopy. Anal intraepithelial neoplasia (AIN) was confirmed by a pathologist [45]. None of them were vaccinated by prophylactic HPV vaccine.

Were excluded from the study participants who did not formally consent to participate and those for whom we failed to have interpretable DNA sequences after the sequencing procedure.

### Sample collection, DNA extraction and genotyping

Genital secretions and anal margin samples were obtained from included women and MSM, respectively. Genital secretions were collected by self-collection method with veil (Veil Collector V-Veil Up UP2™, V-Veil-Up Production SRL, Romania; https://hpv-veil.com), as previously described [46]. Anal samples were collected using a flocked swab (Copan Diagnostic Inc., California, USA). All samples were brought in an ice pack to the virology laboratory of the Hôpital Européen Georges Pompidou, Paris, France. DNA was extracted from the cervical swab and anal specimen using the DNeasyBlood and Tissue kit (Qiagen, Hilden, Germany) as recommended by the manufacturer, eluted in 100μL of the kit elution buffer and store at -20°C before genotyping and sequencing. The genotyping of HPV DNA in treated samples were done as previously described [10, 30], using Anyplex™ II HPV28 detection test as recommended by the manufacturer (Seegene, Seoul, South Korea).

### L1, E2, E6, E7 genes and LCR amplification and sequencing

Specific primers targeting each of the L1, E2, E6, E7 genes and LCR of the HPV35 reference genotype (HPV35H; GenBank accession number: X74477) [47] were designed using the online software Primer3Plus (http://www.bioinformatics.nl/cgi-bin/primer3plus/primer3plus.cgi) and are presented in S1 Table. An alignment analysis using the online sequence alignment tool BLAST (Basic Local Alignment Sequence Tool: https://blast.ncbi.nlm.nih.gov/Blast.cgi) was done to ensure the specificity of the designed primers. A polymerase chain reaction (PCR) assay was carried out in 50μL of final reaction mix using 5μL of DNA from study specimens and 1X GoTaq^®^ DNA Polymerase all-in one optimized PCR master mix (Promega, Madison, Wisconsin, USA), containing 10 mM Tris buffer (pH 8.5); 0.2 mM of each deoxyribonucleotide (dATP, dGTP, dCTP, dTTP); 1.5 mM MgCl_2_, and supplemented with 0.25 μM of each designed set of primers. The thermal cycling amplification algorithm was set as follows: 2 minutes at 95°C, 45 cycles of 30 seconds at 95°C, 30 seconds of hybridization at 55°C (for all sets of primers), followed by an extension step of 60 seconds at 72°C, and a final extension step at 72°C for 8 minutes occurred at the end of the cycling process. A 1% agarose electrophoresis was also performed to ensure the good amplification of the genes.

A PCR Cleanup reaction was carried out in PCR products using 3μL of the PCR Cleanup^®^ kit (CELERA, Almeda, USA) containing the exonuclease I and the alkaline phosphatase to remove all non-amplified nucleic acids (excess of primers, dNTPs, DNA templates), according to the following thermal algorithm: 15 minutes at 37°C followed by 15 minutes at 80°C.

The purified PCR products were then sequenced using the ABI PRISM^®^ BigDye Terminator v1.1 Cycle Sequencing kit as recommended by the manufacturer (Applied Biosystems, Foster City, CA, USA). Briefly, 6μL of purified DNA were added into 16μL of a sequencing master mix containing 3μL of a 5X sequencing buffer (Applied Biosystems); 1μL of BigDye Terminator 94 V1.1/V3.1; 4μL of the same set of primers (2μL for each primer) used for the amplification step and 8μL of nuclease-free water (Promega). The sequencing algorithm was set as follows: 25 cycles of 15 seconds at 94°C, 10 seconds at 55°C, 4 minutes at 60°C followed by an inhibition step at 4°C until subsequent analyses. The sequencing products were purified through a gel filtration medium Sephadex^®^ G-50 (GE Healthcare Life Sciences, Marlborough, MA, USA) and purified DNA sequences were then analyzed by the 3730xl DNA sequencer Analysis (Applied Biosystems). The electropherograms of the obtained sequences were analyzed with Sequencer DNA Sequence Analysis Software^®^ version 5.4.6 (Gene Codes Corporation, Ann Arbor, MI, USA).

### Bioinformatic analyses

The genetic variability in each of the L1, E2, E6, E7 genes and the LCR of study participants was estimated by aligning obtained sequences with L1, E2, E6, E7 open-reading frames and the LCR sequence of the prototype HPV35H, respectively, using the MEGA 7.1 software (http://www.megasoftware.net). To evaluate whether and to what extent the genetic variability correlates with the selective pressure into key genes of HPV35 from study participants, synonymous (dS) and nonsynonymous (dN) nucleotide differences between each pair of L1, E2, E6, E7, and LCR sequences from study participants and those from the HPV35H prototype, corresponding to the genetic distance (d) between paired sequences, were determined using the Nei-Gojobori model [48]. Thus, the nonsynonymous/synonymous ratio rates (ω = dN/dS), giving insight into the selective pressure in the encoded protein, were further calculated for each amino acid sequence deriving from L1, E2, E6, and E7 genes. For this analysis, ω = 1 corresponded to neutral selection, ω < 1.0 purifying selection, and ω > 1.0 diversifying positive selection favoring diversity within proteins encoded by the same gene [49–51].

Concatenated partial viral genome sequences including L1, E2, E6, E7 and LCR were used to analyze the potential phylogenetic relationship between the HPV35 strains circulating in our study population and those circulating in other populations worldwide. A phylogenetic tree was constructed using the MEGA 7.1 software, according to the Neighbor-Joining method of the Tamura-Nei model, and rooted with the concatenated sequence of HPV18 reference prototype (GenBank accession number: X05015).

All the sequences obtained in this study were submitted to the NCBI GenBank database with the following accession numbers: **LCR**: (OR877087-OR877097); **L1**: (OR877098-OR877104); **E7**: (OR877105-OR877115); **E6**: (OR877116-OR877125); **E2**: (OR877126-OR877134).

### Statistical analyses

Means and standard deviations (SD) were calculated for quantitative variables and proportions for categorical variables. Pearson’s χ2 was used for categorical variables. Average genetic distances were compared within each gene between subgroups using the Welch’s t-test, assuming unequal variances between subgroups.

### Ethics statement

This study was formally approved by the Scientific Committee of the Faculty of Health Sciences (“FACSS”) of Bangui, under the following agreement: UB/FACSS/CSCVPER, and the Scientific Committee of the Faculty of Health Sciences of the University of N’Djamena, constituting the National Ethical Committee of Chad. The study was conducted in compliance with the ethical standards of the responsible institution on human subjects as well as with the Helsinki Declaration. All included women gave their informed signed consent to participate to the study. The record of the consent to participate was documented on each questionnaire. This consent procedure was formally approved by the Ethical Committee (Faculty of Health Sciences of the University of N’Djamena). In addition, all MSM participants were of adults and gave their informed oral consent to participate in the study. For each MSM, the record of the consent was documented in each questionnaire. This consent procedure was formally approved by the National Ethical Committee of Bangui. The authors did not have access to information that could identify individual participants during or after data collection.

## Results

### Sociodemographic and clinical characteristics of study population

According to the inclusion criteria, 6 women from Chad and 8 MSM from CAR, positive for cervical and anal HPV35 DNA, respectively, were initially selected. To minimize sequencing errors in our analysis, we systematically excluded DNA sequences with purity yields below 70%. Below this threshold, the sequences were difficult to interpret. Thus, after the sequencing procedure, 1 woman and 3 MSM were excluded because of insufficient interpretable DNA sequences for any of the 5 genome regions of interest (major capsid L1, E2, E6, and E7 genes, and the LCR).

Finally, a total of 10 participants (mean age: 32.9 years; range: 21–46; sex ratio male/female: 5/5), positive for anal or cervical HPV35 DNA and harboring normal and abnormal cytology were recruited among Central African MSM and Chadian women attending the main healthcare centers for sexual health in Bangui in CAR and N’Djamena in Chad, respectively (**Table 1**). MSM were slightly younger (mean age: 28.8 years; range: 21–39) than heterosexual women (mean age: 37 years; range: 26–46). All MSM were infected with HIV-1, and only one woman was positive for HIV-1 infection. Finally, all MSM showed AIN, whereas all women showed normal cervical cytology (**Table 1**).

**Table 1:** Sociodemographic, clinical, and biological characteristics of women and men positive for cervical and anal Human papillomavirus type 35 DNA.

Patient ID	Geographic origin (Country)	Age (years)	Biological sex	HIV status	Sexual behavior	Anatomic origin of the sample	Cytology
**#CAR1**	CAR	39	Male	Positive	MSM	Anal	AIN
**#CAR2**	CAR	30	Male	Positive	MSM	Anal	AIN
**#CAR3**	CAR	21	Male	Positive	MSM	Anal	AIN
**#CAR4**	CAR	30	Male	Positive	MSM	Anal	AIN
**#CAR5**	CAR	24	Male	Positive	MSM	Anal	AIN
**#CHAD1**	Chad	37	Female	Positive	Heterosexual	Endocol	Normal
**#CHAD2**	Chad	33	Female	Negative	Heterosexual	Endocol	Normal
**#CHAD3**	Chad	43	Female	Negative	Heterosexual	Endocol	Normal
**#CHAD4**	Chad	26	Female	Negative	Heterosexual	Endocol	Normal
**#CHAD5**	Chad	46	Female	Negative	Heterosexual	Endocol	Normal

AIN: Anal intraepithelial neoplasia; CAR: Central African Republic; HIV: Human immunodeficiency virus; MSM: Men who have sex with men.

### Neighbor-joining based phylogenetic relationship of HPV35 study specimens

**Fig 1:**
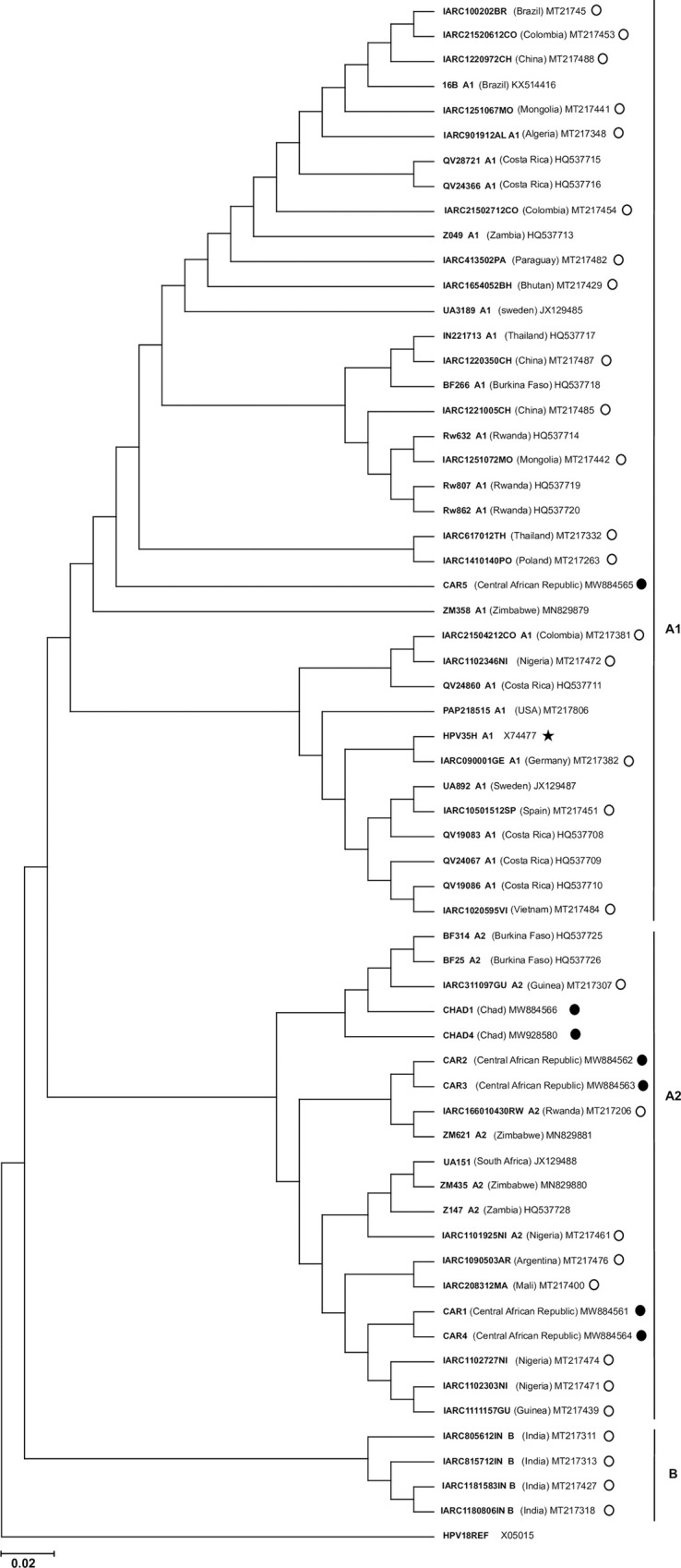
Phylogenetic tree illustrating HPV35 lineages from HIV-infected Central African MSM and HIV-negative heterosexual Chadian women. The molecular phylogenetic analysis was assessed with concatenated partial viral genome including a global alignment of major capsid L1, E2, E6, E7 and LCR nucleotide sequences from (N = 5) anal specimens of HIV-infected Central African MSM and (N = 2) cervical models from HIV-negative heterosexual Chadian women. The phylogenetic tree was inferred using the Neighbor-joining method with 1000 bootstrap replicates. HPV18 was used as an outgroup taxon to root the tree. The identification names of all HPV35 sequences used in this analysis are indicated in bold. HPV35 sequences from the study participants are indicated by uppercase characters “CAR” and “CHAD” followed by the inclusion rank number, the country of origin in parenthesis, the GenBank reference number and a black dot for Central African MSM and Chadian women, respectively. Fifty four (54) HPV35 sequences from specimens of diverse origins were also included in the analysis. For these sequences, the identification name was followed by a space, the lineage or sublineage name (termed A1 or A2, or B), another space, the country where the specimen was isolated put in brackets, another space, and the GenBank accession number. Sequences from HPV35 strains characterized by the International Agency for Research on Cancer (IARC) for HPV35 lineage and sublineage identification were also included in the analysis. The identification name of these sequences starts with “IARC” and is followed by a space, the lineage or sublineage identification, another space, the GenBank accession number, and a white circle. Finally, the HPV35H reference genotype was also included. The identification name of this strain is followed by a space, the sublineage name, another space, the GenBank accession number, and a black star.

The relationship between HPV35 strains isolated from study participants, specimens from the worldwide origin, and the HPV35H prototype genotype was assessed by performing a Neighbor-joining-based phylogenetic tree of the partial concatenated viral genome including the open reading frame (ORF) encoding the major capsid L1, E2, E6, E7, and LCR (**Fig 1**). Globally, the phylogenetic tree could be divided into three main genetic branches containing each sequences belonging to one HPV35 lineage or sublineage. The upper main branch contained HPV35 sequences from the A1 sublineage and could be divided into two other sub-branches, one containing the sequence of the study specimen #CAR5 and the other one containing the sequence of HPV35H reference genotype. Most of the other HPV35 strains from the worldwide origins included in this phylogenetic analysis also fell into in the main branch of the A1 sublineage. The lower main branch was constituted by HPV35 sequences belonging to the A2 sublineage. This branch was also divided into two sub-branches, the upper one containing the study specimen #CAR2, #CAR3, #CHAD1 and #CHAD4 and the lower one containing the study specimen #CAR1 and #CAR4. The third lower branch only included sequences previously characterized by the International Agency for research on Cancer and referred as lineage B (**Fig 1**).

### Genetic diversity and amino acid variations in LCR and L1, E2, E6 and E7 genes of HPV-35 specimens

#### Intra-type diversity in the major capsid L1 gene

Genomic variations within the major capsid L1 gene of HPV35 were determined by pairwise alignment of a 1,483 bp amplicon of L1 gene of HPV35 specimens isolated from study participants. A total of 7 out of 10 HPV35 L1 sequences from 5 Central African MSM (#CAR1; #CAR2; #CAR3; #CAR4 and #CAR5) and 2 Chadian women (#CHAD1 and #CHAD4) were successfully amplified, sequenced, and fully interpretable.

The **Table 2** shows nucleotide variations within the L1 genes of HPV35 strains of study participants compared to the L1 gene of the prototype HPV35H. Globally, 59 nucleotide positions within the L1 gene harbored nucleotide mutations, including 43 nucleotide transversions (72.8%) and 16 nucleotide transitions (27.2%). All L1 sequences showed at least one nucleotide variation compared to HPV35H. The most common mutations represented were G5940A (100%), G6357A (100%), C6501A (100%), G5736A and A7008G (85.7%) (**Table 2**). Viral specimens from Central African MSM harbored higher variability rates (mean number of mutations ± SD: 16±10.8, range: 6–34) compared to Chadian women (mean number of mutations ± SD: 6±1.4, range: 5–7), but without statistical significance (P = 0.113). Specimen #CAR5 showed, however, a significant nucleotide variation of 2.29%, above the nucleotide variation threshold (nucleotide variation rate within the L1 gene above 2%) defining a significant intra-type genetic divergence between an HPV variant and the prototype HPV genotype [52, 53]. Specimen #CAR2 also showed high nucleotide variation rate (1.14%), but this variation remained below the threshold for intra-type genetic divergence. Finally, specimens #CHAD1 and #CHAD4 showed the lowest variation rates (0.47% and 0.33%, respectively).

**Table 2:** Variations of nucleotides in the L1 gene of HPV35 specimens isolated from adult women in N’Djamena, Chad and from MSM in Bangui, Central African Republic.

**HPV** **35**	**Nucleotide positions in L1 gene (1,483bp)**																																																											**Mutations**	**Variability** **(%)**
	**5**	**5**	**5**	**5**	**5**	**5**	**5**	**5**	**5**	**5**	**5**	**5**	**5**	**5**	**5**	**5**	**5**	**5**	**5**	**5**	**5**	**5**	**5**	**5**	**5**	**5**	**5**	**5**	**5**	**5**	**5**	**5**	**6**	**6**	**6**	**6**	**6**	**6**	**6**	**6**	**6**	**6**	**6**	**6**	**6**	**7**	**7**	**7**	**7**	**7**	**7**	**7**	**7**	**7**	**7**	**7**	**7**	**7**	**7**		
	**6**	**6**	**6**	**6**	**6**	**6**	**6**	**6**	**6**	**6**	**6**	**6**	**6**	**6**	**6**	**6**	**6**	**6**	**6**	**6**	**6**	**6**	**6**	**6**	**6**	**6**	**6**	**7**	**8**	**8**	**8**	**9**	**1**	**3**	**3**	**3**	**3**	**3**	**4**	**4**	**4**	**5**	**6**	**6**	**6**	**0**	**0**	**0**	**0**	**0**	**0**	**0**	**0**	**0**	**0**	**0**	**0**	**0**	**0**		
	**1**	**1**	**2**	**2**	**2**	**2**	**2**	**3**	**4**	**4**	**4**	**5**	**5**	**5**	**5**	**5**	**5**	**5**	**6**	**6**	**6**	**6**	**7**	**7**	**8**	**8**	**8**	**3**	**8**	**8**	**9**	**4**	**3**	**3**	**5**	**7**	**8**	**8**	**2**	**4**	**7**	**0**	**4**	**4**	**4**	**0**	**2**	**2**	**2**	**3**	**3**	**4**	**5**	**5**	**6**	**6**	**6**	**8**	**8**		
	**8**	**9**	**0**	**1**	**3**	**4**	**8**	**3**	**2**	**4**	**7**	**0**	**1**	**2**	**3**	**6**	**7**	**8**	**0**	**3**	**7**	**8**	**2**	**3**	**0**	**1**	**2**	**6**	**8**	**9**	**5**	**0**	**8**	**7**	**7**	**1**	**4**	**5**	**4**	**3**	**1**	**1**	**2**	**3**	**4**	**8**	**6**	**8**	**9**	**3**	**4**	**1**	**5**	**9**	**1**	**4**	**6**	**1**	**2**		
**HPV35H**	**C**	**T**	**A**	**A**	**A**	**A**	**C**	**T**	**C**	**C**	**G**	**T**	**C**	**A**	**G**	**T**	**C**	**T**	**A**	**T**	**T**	**A**	**C**	**T**	**T**	**A**	**T**	**G**	**A**	**G**	**G**	**G**	**G**	**T**	**G**	**T**	**T**	**A**	**C**	**C**	**T**	**C**	**T**	**T**	**A**	**A**	**G**	**C**	**T**	**T**	**T**	**A**	**C**	**A**	**C**	**C**	**A**	**T**	**C**		
**#CAR1**																												A				A			A							A			G	G														6	0.40
**#CAR2**	G	C	T																						C	C	A	A	C			A	A		A			T	T			A				G											C		A	17	1.14
**#CAR3**														T														A	C			A	A	G	A							A				G										A		G		11	0.74
**#CAR4**																												A				A			A		A			A	C	A			G	G	A	G	G											12	0.81
**#CAR5**				T	T	T	T	A	A	T	A	G	T	T	A	G	T	G	G	G	A	T	T	G							A	A			A			T				A	C	A						A	A	C	A	G	T					34	2.29
**#CHAD1**																												A		A		A			A	G						A				G														7	0.47
**#CHAD4**																												A				A			A							A				G														5	0.33

HPV35H: Reference prototype viral strain of Human Papillomavirus type 35; A: Adenosine; C: Cythidine; G: Guanosine: T: Thymidine.

#### Amino acid variation in the major capsid L1 protein

**Fig 2:**
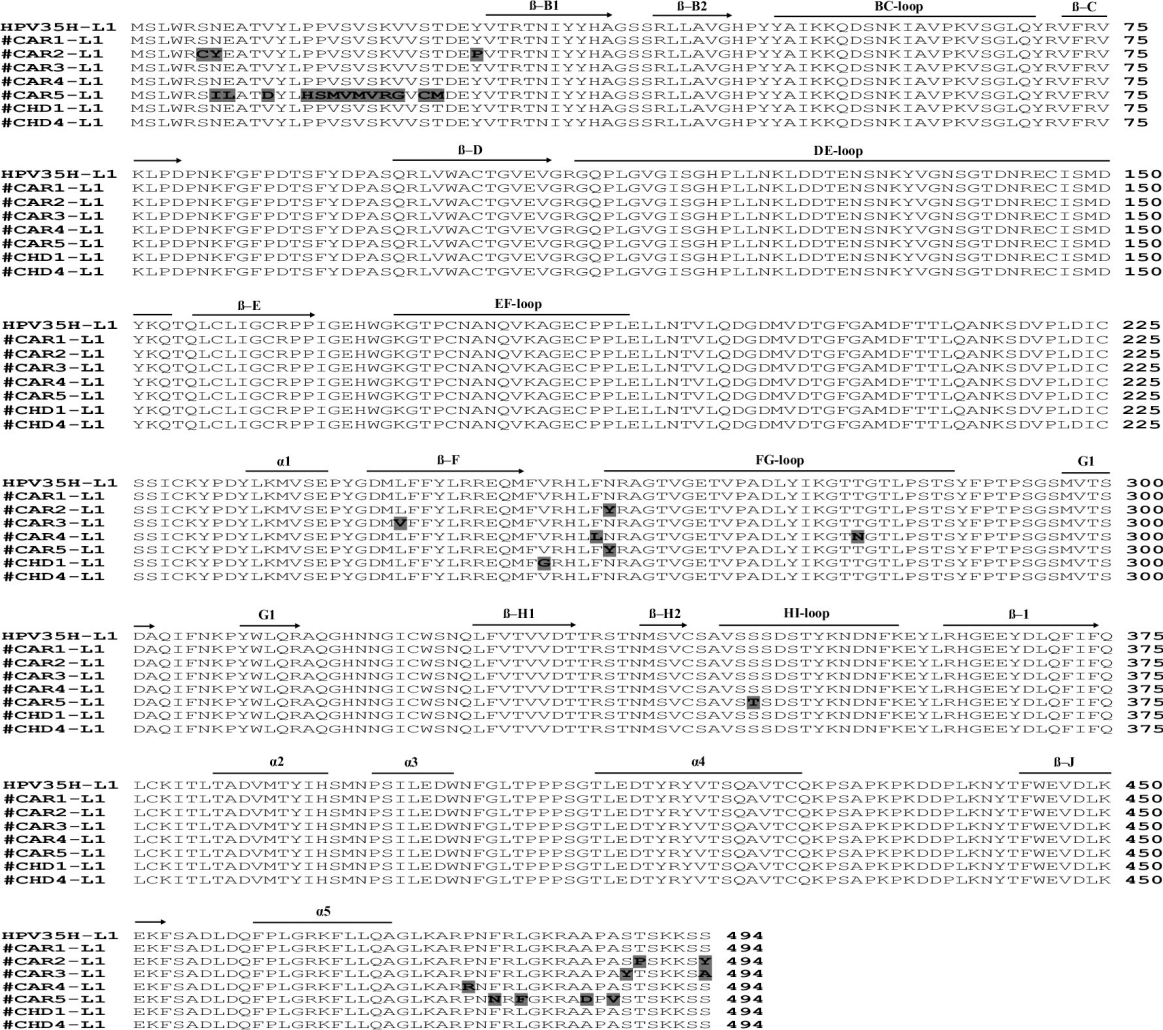
Sequence alignment of L1 from the HPV types HPV35. The residues conserved across the HPV35 specimens are shown in capital letters, whereas the non-conserved residues are highlighted in bold capital letters and dark gray. The β-sheets are shown as arrows. The α-helices are marked as thick bars. The five surface loop domains of the viral capsid are marked thick bars and labeled.

L1 encoding DNA sequences of HPV35 strains isolated from study participants were translated into amino acids and aligned with a 494 amino acids portion of the HPV35H L1 protein, and results are depicted in the **Fig 2**. All viral strains but two (#CAR1 and #CHAD4) exhibited amino acids changes in their L1 protein sequence with an amino acid variability ranging from 0 to 3.84%. Globally, 3 (BC, DE and EF) out of the 5 loops displayed on the surface of the capsid of HPV35 viral particle remained totally conserved, while FG- and HI- loops exhibited amino acid variations. Specimens #CAR2 (N262Y), #CAR4 (T281N) and #CAR5 (N262Y) exhibited amino acids changes within the FG-loop domain and #CAR5 also displayed the S347T within the HI-loop domain. All the 5 α-helices, and 9 out of the 12 β-sheets remained totally conserved, only #CAR3 exhibited the L246V in the β-F sheet. The remaining amino acids variations occurred nearby to the N- and C-terminus of the L1 protein as well as within the peptide portion bounded by the β-F sheet and the FG-loop. Interestingly, #CAR5 exhibited the substitution of 14PPVSVSKV21 by 14HSMVMVRG21 (**Fig 2)**. Remarkably, L1 protein from HPV35 strains isolated from Chadian women remained well conserved, and only #CHAD1 exhibited the V257G (**Fig 2)**.

The analysis of the selection pressure in the L1 gene of HPV35 specimens isolated from study participants was estimated by the ratio (dN/dS) of non-synonymous (dN) to synonymous mutations (dS). Globally, there was a slight negative selection pressure (mean dN/dS = 0.56) within L1 of most clinical specimens suggesting a purifying selection with the maintenance of the prototype L1 gene. However, this purifying selection pressure tended to be higher in specimen isolated from Chadian women (mean dN/dS for cervical mucosa = 0.083) compared to Central African MSM (mean dN/dS for anal mucosa = 0.759), but without statistical significance (*P* = 0.179). Remarkably, the dN/dS ratio of #CAR5 (dN/dS = 2.34) suggested a strong positive and diversifying selection resulting in an HPV35 strain significantly divergent from HPV35H.

#### Intra-type diversity in the LCR

Genomic variations within the LCR were determined by pairwise alignment of the 866 bp amplicon of the LCR of HPV35 viral strains isolated from study participants with the LCR of HPV35H. HPV35 LCR DNA sequence from all the 10 study participants (#CAR1; #CAR2; #CAR3; #CAR4 and #CAR5 from Central African MSM and #CHAD1, #CHAD2, #CHAD3, #CHAD4 and #CHAD5 from Chadian women) were successfully amplified and fully interpretable. The results are shown in **Table 3**.

**Table 3:** Variations of nucleotides in the LCR of HPV35 specimens isolated from adult women in N’Djamena, Chad and from MSM in Bangui, Central African Republic.

**HPV** **35**	**Nucleotide positions in LCR (866bp)**																																											**Mutations**	**Variability (%)**
					**7**	**7**	**7**	**7**	**7**	**7**	**7**	**7**																	**7**	**7**	**7**	**7**	**7**	**7**	**7**	**7**	**7**	**7**	**7**	**7**	**7**	**7**	**7**		
					**1**	**1**	**1**	**1**	**2**	**3**	**3**	**4**																	**4**	**4**	**5**	**5**	**6**	**6**	**7**	**7**	**7**	**7**	**8**	**8**	**8**	**8**	**8**		
	**1**	**2**	**3**	**7**	**1**	**1**	**3**	**7**	**0**	**1**	**3**	**1**																	**1**	**8**	**5**	**6**	**3**	**5**	**0**	**3**	**5**	**7**	**2**	**5**	**5**	**6**	**6**		
	**6**	**4**	**7**	**9**	**7**	**8**	**2**	**9**	**6**	**6**	**4**	**2**																	**3**	**0**	**1**	**9**	**4**	**5**	**8**	**0**	**8**	**6**	**1**	**3**	**9**	**4**	**5**		
**HPV35H**	**C**	**G**	**C**	**A**	**A**	**A**	**A**	**T**	**T**	**A**	**G**	T	-	-	-	-	-	-	-	-	-	-	-	-	-	-	-	-	G	**T**	**T**	**T**	**C**	**G**	**A**	**A**	**A**	**A**	**A**	**C**	**A**	**A**	**G**		
**#CAR1**	A	A											-	-	-	-	-	-	-	-	-	-	-	-	-	-	-	-			C						G		C	T				6	0.69
**#CAR2**	A	A					C						-	-	-	-	-	-	-	-	-	-	-	-	-	-	-	-			C	C							C		T			7	0.80
**#CAR3**	A	A					C						-	-	-	-	-	-	-	-	-	-	-	-	-	-	-	-			C	C							C		T			7	0.80
**#CAR4**	A	A		T									-	-	-	-	-	-	-	-	-	-	-	-	-	-	-	-			C						G			T	T			7	0.80
**#CAR5**			**A**		**C**	**-**		**A**	**A**				**T**	**C**	**T**	**A**	**C**	**C**	**T**	**C**	**C**	**A**	**T**	**T**	**T**	**T**	**G**	**T**		**-**	**C**			**A**			**G**					**T**	**A**	12	1.38
**#CHAD1**	A	A								G	A		-	-	-	-	-	-	-	-	-	-	-	-	-	-	-	-			C		T		T			C		T	T			10	1.15
**#CHAD2**	A	A											-	-	-	-	-	-	-	-	-	-	-	-	-	-	-	-			C						G		C	T	T			7	0.80
**#CHAD3**													-	-	-	-	-	-	-	-	-	-	-	-	-	-	-	-			C													1	0.11
**#CHAD4**	A	A											-	-	-	-	-	-	-	-	-	-	-	-	-	-	-	-			C						G			T				5	0.57
**#CHAD5**	A	A								G	A		-	-	-	-	-	-	-	-	-	-	-	-	-	-	-	-			C					C	G		C	T	T			10	1.15
**TFBS**												**YY1**																									**YY1**								
																										

HPV35H: Reference prototype viral strain of Human Papillomavirus type 35; A: Adenosine; C: Cythidine; G: Guanosine: T: Thymidine; LCR: Long

Control Region; YY1: Ying Yang 1. TFBS: Transcription factor binding sites; (-): deletion.

All study specimens (100%) exhibited at least one nucleotide mutation in the LCR sequence. A total of 26 nucleotide positions within the LCR harbored nucleotide mutations, including 17 (65.4%) transversions, the T7551C (100%) was the most represented, followed by C16A and G24A (80%), A7758G and C7853T (60%), A7821C (50%). Specimen #CAR5 harbored the highest number of mutations (n = 12 mutations), followed by #CHAD1 and #CHAD5 (n = 10). Furthermore, #CAR5 harbored both a 16bp insertion (TCTACCTCCATTTTGT) at position 7412 and deletion at position 7480, both located at binding sites of the transcription factor YY1, and deletion at position 7118 (**Table 3)**.

#### Intra-type diversity in the E2 gene

Out of the 10 participants included, 9 provided fully interpretable 1,101bp sequences of E2 gene, including all the 5 Central African MSM (#CAR1; #CAR2; #CAR3; #CAR4 and #CAR5) and 4 Chadian women (#CHAD1, #CHAD3, #CHAD4 and #CHAD5). Results of the genetic variability of the E2 gene are summarized in the **Table 4**. Each HPV35 harbored at least 5 mutations (mean: 10.5±5.1; range: 5–20) in the E2 gene, and the most represented were C2981A, C3236A, A3476G and A3688C (100%), followed by T3148C (77.8%). The remaining mutations were less frequent (50%). The following one-nucleotide deletion events (del) occurred only in specimens from Chadian women, C2756del in #CHAD1, A3037del and A3140del in #CHAD3, and A3140del and A3257del in #CHAD4. Specimens isolated in Chadian women showed a significant higher rate of genetic variability (mean number of mutations: 15.3±3.7; range: 11–20) in the E2 gene compared to Central African MSM (mean number of mutations: 6.8±1.1; range: 5–8), (*P* = 0.0179). Specimen #CHAD1 and #CHAD5 showed the highest nucleotide variation rates (1.81% and 1.45%, respectively).

**Table 4:** Variations of nucleotides in the E2 gene of HPV35 specimens isolated from adult women in N’Djamena, Chad and from MSM in Bangui, Central African Republic.

**HPV 35**	**Nucleotide positions in E2 gene (1,101bp)**																																						**Mutations**	**Variability (%)**
	**2**	**2**	**2**	**2**	**2**	**2**	**2**	**2**	**2**	**2**	**2**	**2**	**2**	**2**	**2**	**3**	**3**	**3**	**3**	**3**	**3**	**3**	**3**	**3**	**3**	**3**	**3**	**3**	**3**	**3**	**3**	**3**	**3**	**3**	**3**	**3**	**3**	**3**		
	**7**	**7**	**7**	**7**	**7**	**7**	**7**	**7**	**7**	**7**	**7**	**7**	**7**	**8**	**9**	**0**	**1**	**1**	**1**	**2**	**2**	**3**	**3**	**3**	**4**	**4**	**4**	**4**	**6**	**6**	**6**	**6**	**7**	**7**	**8**	**8**	**8**	**8**		
	**2**	**3**	**4**	**5**	**5**	**5**	**6**	**6**	**6**	**7**	**7**	**8**	**8**	**0**	**8**	**3**	**4**	**5**	**5**	**3**	**5**	**7**	**7**	**8**	**4**	**5**	**7**	**8**	**3**	**4**	**8**	**9**	**4**	**9**	**0**	**1**	**1**	**1**		
	**9**	**6**	**3**	**3**	**6**	**7**	**4**	**6**	**9**	**5**	**7**	**3**	**8**	**9**	**1**	**7**	**0**	**3**	**8**	**6**	**7**	**6**	**8**	**8**	**2**	**2**	**6**	**1**	**6**	**8**	**8**	**4**	**0**	**9**	**5**	**0**	**2**	**8**		
**HPV35H**	**T**	**C**	**G**	**G**	**C**	**A**	**T**	**G**	**C**	**G**	**G**	**T**	**C**	**A**	**C**	**A**	**A**	**C**	**T**	**C**	**A**	**T**	**C**	**G**	**C**	**T**	**A**	**C**	**A**	**T**	**A**	**A**	**A**	**A**	**T**	**A**	**G**	**T**		
**#CAR1**	G														A				C	A						C	G				C								7	0.63
**#CAR2**															A				C	A				A			G	T			C								7	0.63
**#CAR3**															A				C	A				A			G	T			C								7	0.63
**#CAR4**	G														A				C	A						C	G				C	T							8	0.72
**#CAR5**	G														A					A							G				C								5	0.45
**#CHAD1**	G		A	T	-	G		A		T	C		G	T	A				C	A						C	G				C	T	C	C		G			20	1.81
**#CHAD3**										A		A		T	A	-	-			A		C	A				G		G	A	C	T							14	1.27
**#CHAD4**			A											T	A		-		C	A	-						G				C					G	C		11	0.99
**#CHAD5**		A					G	A	T				G	T	A			G	C	A					T	C	G				C				G			C	16	1.45

HPV35H: Reference prototype viral strain of Human Papillomavirus type 35; A: Adenosine; C: Cythidine; G: Guanosine: T: Thymidine;; (-): deletion.

#### Amino acid variation in E2 protein

The alignment of the 367 amino acid of E2 protein from the 9 HPV35 specimens with interpretable DNA sequences is presented in the **Table 5**. Globally, all the 9 strains exhibited at least the following 3 mutations (S89R, T171N and Y251C). The remaining mutations were less frequent (50%). Specimens from Chadian women exhibited significantly more mutations (mean number of mutations: 12.0±4.24; range: 7–16) in the E2 protein Central African MSM (mean number of mutations: 5.6±1.81; range: 3–8), (*P* = 0.036). While #CHAD1 and #CHAD5 exhibited the highest rates of variability (4.35% and 4.08%, respectively), #CAR5 (0.81%) was the less impacted specimen.

**Table 5:** Amino acid variations in the E2 protein of HPV35 specimens isolated from adult women in N’Djamena, Chad and from MSM in Bangui, Central African Republic.

HPV35	Amino acid positions in E2 protein (367AA)																															Mutations	Variability (%)
														1	1	1	1	1	1	2	2	2	2	2	2	3	3	3	3	3	3		
		1	1	1	1	1	1	1	2	2	2	3	8	0	3	4	4	7	7	1	2	4	4	5	5	2	3	5	6	6	6		
	8	0	3	4	5	7	8	9	1	3	5	2	9	5	9	3	5	1	8	8	2	0	3	1	3	4	9	9	1	3	5		
HPV35H	R	S	Q	D	K	L	E	H	E	D	T	Q	S	T	Y	D	I	T	K	Y	E	H	L	Y	P	T	Y	T	S	G	M		
**#CAR1**													R				T	N					P	C								5	1.36
**#CAR2**													R				T	N			K			C	S							6	1.63
**#CAR3**													R				T	N			K			C	S							6	1.63
**#CAR4**													R		-		T	N	-				P	C		S						8	2.17
**#CAR5**													R					N						C								3	0.81
**#CHAD1**		N	H	-	E		K		Y		R	L	R				T	N					P	C		S	S	P				16	4.35
**#CHAD3**									K	E		L	R	-	-			N		Q				C		S						10	2.72
**#CHAD4**		N										L	R				T	N						C						A		7	1.90
**#CHAD5**	S					R	K	Y			R	L	R			E	T	N				Y	P	C					A		T	15	4.08

HPV35H: Reference prototype viral strain of Human Papillomavirus type 35; AA: Amino acid; A: Alanine; C: Cysteine; D: Aspartic acid; E: Glutamic acid; F: Phenylalanine; G: Glycine; H: Histidine; I: Isoleucine; K: Lysine; L: Leucine; M: Methionine; N: Asparagine; P: Proline; Q: Glutamine; R: Asparagine; S: Serine; T: Threonine; Y: Tyrosine.

Globally, there was a positive selection pressure (mean dN/dS = 5.09±4.1) within the E2 gene of most clinical specimens suggesting a purifying selection with the maintenance of the prototype E2 gene. Specimen isolated in women showed higher response against selection pressure (mean dN/dS = 6.7±5.6) in the E2 gene compared to HIV-infected MSM (mean dN/dS = 3.8±2.1), but without a statistical significance (*P* = 0.389).

#### Intra-type diversity in the oncoproteins E6 and E7

Regarding the E6 gene, all the 10 participants included provided a fully interpretable 447bp of the E6 gene. Overall, the E6 gene of all the 10 HPV35 strains appeared well conserved and only 8 positions were affected by a nucleotide substitution. Two significant substitutions (C131A and A295T) were found in all the 10 specimens, followed by T127C, G128C, T136C, T229C, A313G, and T341C, which were seeding once in the total of the specimens. #CAR5 was the most variable specimen, which exhibited a significant degree of divergence (1.12%).

The E7 gene of the HPV35 strains of the study participants were very well conserved. Only the following two mutations (T114C and G187A) were present. While the mutation T114C was present in 6 specimens, including 4 Central African MSM (#CAR1, #CAR2, #CAR3, and #CAR4) and 2 Chadian women (#CHAD1 and #CHAD5), the mutation G187A was found only in #CHAD5. E7 genes #CAR5, #CHAD2, #CHAD3 and #CHAD4 remained all totally conserved.

#### Amino acid variation in the oncoproteins E6 and E7

Like what was observed in the DNA sequences of the E6 gene, the 149 amino acid chains of the E6 oncoprotein of the study specimens remained well conserved. Only the mutations E7Qin #CHAD3 and W78N in #CAR5 were present.

Regarding E7 protein, all but one (#CHAD5) amino acid sequences remained totally conserved. Only the E63K was observed in #CHAD5.

## Discussion

We herein report the intra-genotype diversity in L1, E2, E6, and E7 genes and the LCR of HPV35 specimens isolated from cervical mucosa of HIV-negative heterosexual adult women with normal cervical cytology living in N’Djamena, Chad, and from anal mucosa of HIV-infected MSM with anal intraepithelial neoplasia living in Bangui, CAR. Overall, we observed a high rate of silent natural polymorphism mutations in L1, E2, E6, E7, and LCR of most of the study specimens. The phylogenetic analysis inferred on concatenated sequences including L1, E2, E6, E7 and LCR ORF reveals that most of the HPV35 study specimens clustered into the A2 sublineage, independent of the anatomical sites of sampling. Only one study specimen belonged to the A1 sublineage as the HPV35 reference genotype (HPV35H). Interestingly, this HPV35 specimen (#CAR5) isolated from the anal mucosa of an HIV-infected Central African MSM exhibited a significant high rate of variability (2.29%) in the L1 coding sequence, indicating that this individual would be infected by a genetic variant potentially divergent from the prototype HPV35. This HPV35 specimen also harbored a higher rate of genetic variability in the LCR and E6 oncogenes, while its E7 sequence was totally conserved. The strong variability of the HPV35 specimen #CAR5 could significantly impact the pathogenicity of this viral strain by increasing or decreasing its viral fitness. Despite the particularity of specimen #CAR5, these observations demonstrated the relative maintenance of a well conserved HPV35 A2 sublineage within two populations with different sexual behaviors, heterosexual women living in Chad and MSM living in the CAR.

HPV variant lineages represent further evolutionary divergence but also differ in the cancer risk [33, 42, 52, 54], making it interesting to evaluate their distribution within at-risk populations. In our study, the phylogenetic analysis of a concatenated partial viral genome revealed that almost all HPV35 specimens isolated from the cervical mucosa of Chadian women as well as from the anal mucosa of HIV-infected Central African MSM clustered into the A2 sublineage. Only one specimen isolated from the anal mucosa of an HIV-infected MSM belonged to the A1 sublineage as the HPV35 reference genotype. Contrary to the high genetic variation observed between variants of other high-risk HPV types [54], the low phylogenetic distance pattern between HPV35 variants presented here is consistent with what is commonly observed in other populations around the world, with most HPV35 variants clustering between only two sublineages (A1 or A2 sublineage) belonging both to only one lineage (lineage A) [33, 42, 52, 54]. The recent evolutionary divergence of the HPV35 genotype from the common ancestor of the HPV16-related genotypes clade may explain the singular phylogenetic pattern observed between its variants [54]. Only very little is known about the relationship between HPV35 genetic variability and the risk of HPV-associated cancer [33, 42, 52, 54], particularly anal cancer in MSM for whom associated data do not yet exist, to our knowledge.

The link between HPV35 lineage and the risk of cervical cancer in women is likely to vary depending on the infected population and ethnicity [33, 43, 55]. In our study population, HPV35 specimens from Chadian women with available L1 DNA sequences all fell into the A2 sublineage branch of the phylogenetic tree. Our findings are consistent with the distribution reported from a large population-based study, including HPV35 specimens from women of worldwide origin and from multiple ethnic groups, and showing that HPV35 specimens belonging to the A2 sublineage were more frequently detected in women living in African regions [33]. These singular distribution patterns of the HPV35 A2 sublineage would suggest a possible co-evolutionary adaptation of this sublineage with the African population. These findings are of great interest when considering the high burden of HPV35-associated cervical cancers in Africa and in people with African ancestry [13, 19, 33–36]. Indeed, a study carried out on women from Guanacaste in Costa Rica, a region populated by a majority of European descendants [56], found that the A1 sublineage was significantly associated with a higher risk of persistence and the development of CIN3+ than the A2 sublineage [43].

Another study from the United States of America (USA) (from which we were not able to find the ethnic distribution) did not find any difference in the risk of high-grade lesions, persistency, or even cervical cancer between these two sublineages [55]. In contrast, the study from Pinheiro et al. demonstrated that the A2 sublineage was significantly more associated with CIN2+ and CIN3+ in black American women compared to other ethnic groups living in the USA [33]. In addition, the A2 sublineage was responsible for a significantly higher proportion of intraepithelial cervical carcinoma (ICC) and high-grade cervical lesions in women living in Africa compared to any other regions throughout the world [33]. Furthermore, A2 sublineage was inversely associated with high-grade lesions in white women, and A1 sublineage was found to be significantly more frequent than A2 sublineage in non-African world regions for ICC and high-grade lesions [33]. A similar distribution was also reported in women living in Zimbabwe in whom HPV35 specimens belonging to A2 sublineage were detected only in women with high-grade cervical lesions [42]. This particular association may suggest an increase in the evolutionary fitness of the A2 sublineage within the African population or those with African ancestry, making it possible to escape the host’s immune system and thereby allowing it to persist and ultimately induce cancer. According to these findings, although all women in our study showed normal cytology and were free of the other as well as most carcinogenic, high-risk HPV genotypes such as HPV16 or HPV18, they still present a considerable risk of developing high-grade lesions and even cervical cancer, as previously observed in other African settings, such as South Africa [22]. Future larger viral genomic studies are warranted, especially to identify the genetic basis for HPV35 A2 sublineage’s unique distribution pattern and carcinogenicity in African women.

Regarding the Central African MSM, our findings constitute the first report, to our knowledge, on the phylogenetic distribution of HPV35 specimens isolated from the anal mucosa of a MSM population. We found that four out of the five HPV35 specimens isolated from the anal mucosa of the Central African MSM clustered in the A2 sublineage, and only one belonged to the A1 sublineage. It is possible, as for women, that the A2 sublineage represents the major sublineage circulating in the MSM population in the CAR. Indeed, such association between HPV lineage and an ethnic group was previously reported for HPV16, with the B lineage showing a higher risk for inducing anal cancer in black American men compared to other ethnic groups, therefore suggesting a stronger carcinogenic potential in this African American population [57]. However, further larger genomic studies are needed to confirm or affirm this assumption. Finally, we were not able to highlight any specific distribution of HPV35 sublineage according to the anatomic site (*i*.*e*., anus *versus* cervix) or HIV infection.

Regarding the genetic variability, L1 DNA sequences from all HPV35 specimens harbored a frequency of mutations ranging from 0.33% to 2.29%, corresponding mostly to natural silent polymorphism mutations eliciting only a low level (ranging from 0 to 3.84%) of amino acid changes in the major capsid L1 protein. In addition, major domains of the L1 protein, such as capsid surface loops (BC, DE, and EF) containing main immunodominant neutralizing epitopes, as well as the secondary structures α-helices and β-sheets remained, for most of them, well conserved. Our findings are consistent with previous studies reporting that the major capsid L1 protein of HR-HPV genotypes, including HPV35, remained well conserved despite the high polymorphism within the L1 gene [58–60]. On the other hand, L1 protein from HPV35 specimens isolated from the anal mucosa of 3 MSM exhibited amino acid changes in either the FG or the HI surface loop domains. Moreover, one of them (#CAR5) exhibited amino acid alterations in both FG and HI surface loops. Alterations within immunodominant conformational epitopes such as FG and HI domains may affect the infectivity and pathogenicity or alter the antigenicity of these viral specimens. For HPV16 and other closely related genotypes, a single mutation in the FG or HI domain of the L1 protein was sufficient to affect the recognition of the virus by both type-specific neutralizing as well as cross-reactive antibodies [58, 61, 62]. Another report also showed that FG and HI surface loops of HPV16 L1 protein are significantly involved in human sulfate-heparan binding, and amino acid alterations in those domains significantly reduced the viral infectivity of HPV16 pseudovirus *in vitro* [63]. As several residues of these two domains are well conserved between HPV16 and HPV35 L1 proteins [64], we could speculate that the alterations observed in our study may similarly alter the infectivity of these viral specimens. The N- and C-terminal domains of the L1 protein of HPV35 specimens from study MSM were strongly affected by amino acid changes. These two domains have been shown to significantly contribute to the viral assembly of the HPV capsid and its immunogenicity (N-terminus) [65–67] and also the viral-host interactions (C-terminus) [68]. Therefore, the alterations observed in these two domains in the L1 proteins of HPV35 specimens from the MSM population may have an impact on the viral strains fitness by significantly reducing their ability to self-assemble into a functional viral capsid able to bind to cellular receptors, thereby hampering their uptake into the target cells [63]. Remarkably, the high variability rate (2.29%) detected in the L1 DNA sequence from a Central African MSM specimen (#CAR5) likely suggests that this individual would likely be infected by a genetic variant potentially divergent from the prototype HPV35 genotype. Indeed, according to the traditional criteria used to classify HPV, a minimum of 2% dissimilarity in the L1 DNA sequences between the prototype HPV type and a given clinical specimen is sufficient to define a divergent variant [52, 53, 69]. Such divergence between the HPV35 specimen (#CAR5) and the prototype HPV35 strain could have a significant impact on the pathogenicity of this potential variant by increasing or decreasing its viral fitness. A whole-genome analysis is necessary for this specimen to definitively conclude whether a divergent variant of HPV35 is circulating within this population of MSM living in Central Africa and to define the potential epidemiological and clinical implications. The Central African populations are basically resident and landlocked within the center of the African continent with no or very few migrations. The human population bottlenecks may have negatively impacted the evolutionary potential of HPV35 and allowed the concentration of new and divergent strains of HPV35 not yet reported in the rest of the world. The hypothesis of the concentration of unique strains of HPV35 in homosexual MSM minority populations particularly excluded from the dominant heterosexual population in Central Africa can also be raised. Currently available prophylactic vaccines do not target HPV35. Nevertheless, the existence of divergent variants of HPV35 could become an additional obstacle to the actualization of prophylactic HPV vaccine in which new targeted HPV genotypes could have been added.

Consistent with the previous reports on the genetic variability of the HPV35 LCR sequences, we have found similar rates of mutations ranging from 0.11% to 1.38% in the LCR sequences of the study specimens, without any significant difference between the isolates from Chadian women and Central African MSM [52, 69–71]. The HPV35 specimen (#CAR5), which exhibited the highest mutation rate in the L1 sequence, also showed the highest variability in the LCR sequence. Interestingly, only this specimen exhibited the well-described 16bp insertion between nucleotides T7412 and G7413 [42, 52, 70] and the nucleotide transversion A7758G [52, 69–71], both located at binding sites of the YY1 transcription factor [69], and also the deletion of the thymidine at position 7480 [52, 69, 71]. These observations are of critical relevance as mutations within this regulatory region influence the replication of the HPV viral genome and the transcription of genes encoding the oncoproteins E6 and E7 through their effect on regulatory protein complex formation on DNA [72]. Such a mutation pattern could enhance the pathogenicity of the HPV35 specimen #CAR5. It has been reported that nucleotide changes occurring at YY1 binding sites of HPV16, which is the HPV35-closely related genotype, would be part of the mechanisms involved in the overexpression of the oncoprotein E6 and E7 during the course of cancer progression [72, 73].

Besides L1 and LCR sequences, we further evaluated the genetic variability within early genes E2, E6, and E7. We herein report significant variability of the E2 gene encoding the regulatory early protein 2 of HPV35 specimens isolated in two different anatomic sites (cervical and anal mucosa) from two different populations (Chadian women and HIV-infected Central African MSM). HPV35 specimens isolated from Chadian women exhibited the highest variability in both E2 DNA and amino acid sequences compared to viral specimens isolated from HIV-infected Central African MSM. E2 protein is the main transcriptional regulator of HPV genes, and the interaction of E2 protein with several host cellular proteins, such as the Bromodomain-containing protein 4 (Brd4) in infected cells, allows the repression of E6 and E7 genes [74]. Non-synonymous mutations affecting the binding domains of cellular factors in E2 protein could inhibit the stability of the E2-cellular factor forming complex and lead to the alleviation of the repression of E6 and E7 genes, thereby inducing the overexpression of the oncoproteins E6 and E7 [74, 75], and contributing to the progression toward malignancy [74]. The high rate of non-synonymous mutations observed in the E2 protein of HPV35 specimens from Chadian women could affect the stability of these viral specimens and favor their oncogenicity. Given the importance of the E2 protein in the process of malignancy and the high burden of HPV35-associated cancers in the African population, it would be necessary to further elucidate the impact of such high non-synonymous mutation rates with functional studies. Regarding the oncogenes, although the E6 gene exhibited a total of 7 mutations, among which 6 (T127C, C131A, T136C, A295T, A313G, and T341C) were previously described [71], and E7 exhibited two mutations, most of them were synonymous mutations, with no significant changes in the E6 and E7 oncoproteins.

It is obvious that our study has some limitations, including mostly the small sample size of the study, and the fact that our estimations of the genetic diversity of HPV35 are mainly based on selected genes and not on the whole genome variability. Additionally, a larger group of contextual sequences from Central Africa collected prospectively in the different group of populations would have been ideal for better appreciating individual genetic diversity and the associated effect on the pathogenicity of HPV35 variants circulating in this region. Moreover, these larger prospective studies would help to understand how these particular variants circulate within the different group of central African men and women, and to what extent the migrations of these populations toward other continent hosting other HPV genotypes could affect the HPV epidemiology in those regions.

In conclusion, our genetic analysis highlights the relative maintenance of a well conserved HPV35 A2 sublineage within two populations with different sexual behaviors, heterosexual women living in Chad and MSM living in the CAR. A whole-genome analysis would be helpful to better characterize the likely divergent HPV35 specimen isolated in the MSM population. Moreover, larger prospective series of HPV35 positive women or MSM are warranted to better understand the clinical impact of this genotype in the African population. This information would help improve the prevention strategies already in place on the African continent, making them more specific to the African epidemiological context.

## Supporting information

S1 Checklist STROBE statement—checklist of items that should be included in reports of observational studies. (DOCX)

S1 Table Specific primers targeting each of the L1, E2, E6, E7 genes and LCR of the HPV35 reference genotype. (DOCX)
