# Supplementary material for: Genetic diversity of HPV35 in Chad and the Central African Republic, two landlocked countries of Central Africa: A cross-sectional study
Source: PLoS One. 2024 Jan 25;19(1):e0297054. doi: 10.1371/journal.pone.0297054 (PMC10810494; doi:10.1371/journal.pone.0297054)
Supplement: S1 Table — (DOCX) [file pone.0297054.s002.docx]

**Table S1.** Characteristics of the specific primers targeting L1, E2, E6, E7 and LCR of the HPV35 reference genotype (HPV35H; GenBank accession number: X74477).

| **Target** | **Primer sequences**  **(5'🡺3')** | **Tm**  **(°C)** | **Amplicon length**  **(bp)** |
| --- | --- | --- | --- |
| **L1-HPV35-forward** | CGTAAACGTATCCCATATTT | 55.6 | 1559 |
| **L1-HPV35-reverse** | TACATGCATACACATTTACAC | 51.4 |  |
| **LCR-HPV35-forward** | AATCTTCTACTAAACGTAGAAAAG | 53.9 | 948 |
| **LCR-HPV35-reverse** | CAGTTTGTAAGGTCGTTCAG | 57.4 |  |
| **E2-HPV35-forward** | GATAAAAATGGAAACCCAGT | 56.9 | 1324 |
| **E2-HPV35-reverse** | CTGAGTATAATGACACAGATAG | 50.5 |  |
| **E6-HPV35-forward** | GAAGTGGACAGACATTGTAA | 54.9 | 570 |
| **E6-HPV35-reverse** | GTCACACAATTGCTCATAAC | 55.4 |  |
| **E7-HPV35-forward** | AAAACGATTCCATAACATCG | 58.9 | 549 |
| **E7-HPV35-reverse** | TATAAAGTCCACCATATCCTC | 54.7 |  |
